# Understanding Physician Attitudes Toward AI in Clinical Decision-Making: Cross-Sectional Study

**DOI:** 10.2196/79730

**Published:** 2025-12-08

**Authors:** Fahad Alhazmi

**Affiliations:** 1 Department of Health Services and Hospitals Administration, Faculty of Economics and Administration, King Abdulaziz University, Jeddah, 21589, Saudi Arabia, 966 507088878

**Keywords:** artificial intelligence, AI, health care technology, physicians, perception, barriers, solutions

## Abstract

**Background:**

The Kingdom of Saudi Arabia (KSA) has made tremendous efforts to promote the adoption of advanced technologies such as artificial intelligence (AI). While the successful adoption of AI is dependent on physician perception, there is a scarcity of data concerning KSA physicians’ perception of the technology.

**Objective:**

The purpose of this study was to conduct a cross-sectional survey that would provide updated statistics on physicians’ attitudes toward AI with a focus on ethical and practical perspectives among physicians licensed in the KSA.

**Methods:**

A pilot study was conducted with 10 physicians to enhance the clarity of the survey questions. The pilot was followed by a cross-sectional survey, which was conducted through 25 online, self-administered questionnaires hosted on Qualtrics. A total of 218 physicians filled out the survey. The dataset was then exported into Microsoft Excel and analyzed using descriptive statistics rather than inferential analyses given the exploratory nature of this study and its primary aim to generate updated descriptive insights rather than test specific hypotheses.

**Results:**

A total of 201 fully filled surveys, representing 127 (63.2%) female and 74 (36.8%) male physicians with experience ranging from 3 to ≥30 years, were analyzed. Most physicians (n=165, 82.1%) trusted AI-based clinical decision-making, and 76.6% (n=154) believed that the technology improved efficiency in health care delivery. Unfortunately, only 25.9% (n=52) of physicians had used AI in the previous year. Common barriers to AI adoption included lack of training, high implementation costs, and resistance to change, as well as concerns related to privacy, data security, bias in AI-based recommendations, patient autonomy, and liability. Participants recommended training through workshops (n=50, 25%), online courses (n=47, 23.4%), hands-on experience (n=44, 21.9%), and a combination of online courses and hands-on experience (n=17, 8.5%).

**Conclusions:**

Physicians who responded to this survey supported AI’s use in health care but reported facing financial, ethical, and training barriers, which could be addressed through informed consent and staff training.

## Introduction

### Overview and Background

Artificial intelligence (AI) is an emerging technology that has gained widespread popularity in various sectors. AI is described as a powerful and disruptive technology that facilitates the use of computing devices and augmented systems to simulate critical thinking and intelligent behavior with the help of machine learning [1]. The Kingdom of Saudi Arabia (KSA) is among the high-income countries that have made efforts to tap into the power of AI in health care. Notably, the kingdom is currently undergoing substantial health care reforms that include embracing new technological advancements such as AI, whose integration is considered critical toward achieving its goals [2]. In this case, the adoption of AI aligns with the KSA’s emphasis on the integration of technological advancements and innovation and is consistent with the Ministry of Health’s support for AI-driven projects and efforts to pilot AI applications in clinical settings through collaboration with both local and international technology firms [2]. The KSA’s commitment to AI is evident in initiatives such as Seha Virtual Hospital—currently the world’s largest virtual hospital—which have tapped into the power of AI to overcome geographical barriers and resource constraints in the delivery of care [3,4]. Similarly, King Faisal Specialist Hospital and Research Centre has demonstrated the kingdom’s commitment to AI with its development of several AI applications intended to contribute toward the goal of leveraging the power of AI to improve health outcomes through the Vision 2030 goals [2,5]. To reaffirm its commitment to AI and other advanced technologies, the KSA has established the Saudi Data and AI Authority, which establishes governance in AI and data in collaboration with critical sectors such as education and health care [6]. Despite these efforts by various stakeholders in the KSA’s health care sector, successful implementation of AI heavily depends on positive perception among physicians and other clinicians. The aim of this study was to explore physicians’ attitudes toward AI with a focus on ethical and practical perspectives among physicians licensed in the KSA.

### Problem Statement

There is a growing consensus among scholars that successful implementation of AI is dependent on scholars’ perceptions and acceptance of this technology [7-9]. Recent studies suggest that, while many health care professionals in the KSA acknowledge AI’s potential benefits, concerns persist regarding the impact of this technology on patient safety and quality health outcomes, job displacement, data reliability, and the ethical implications resulting from AI-driven decision-making. In terms of perception and acceptance, the KSA’s physicians have shown a polarized attitude toward AI and its adoption in health care. For instance, professional health care organizations such as the American Heart Association have endorsed AI technologies such as ChatGPT after finding that the technology is 84% accurate, but the same technology has failed the accuracy test in some Asian countries, such as Taiwan [10]. At the local level, 76% of physicians in Jeddah, KSA, agreed that AI was accurate, and 69% of these participants agreed that the technology enhanced efficiency in health care delivery [8]. Surprisingly, only 25.9% reported using this technology in the previous year [8]. Similarly, 61.6% of physicians from the KSA who used AI agreed that the technology was useful, whereas 62.7% and 61.8% agreed that it enhanced efficiency and lowered the cost of care, respectively [7]. However, one study found that the adoption of AI was relatively higher among KSA physicians compared to non-KSA physicians at 55.7% versus 44.3% [7]. A study reported that 61.1% of KSA physicians expressed a positive perception of the integration of AI in health care. These physicians also cited ethical concerns such as job replacement, data privacy, and concerns about the accuracy of AI systems in clinical decision-making [7-9,11]. In contrast, one study found that only 26.2% of KSA physicians had a positive perception of AI and that only 12.3% of these professionals had the technical expertise to use these technologies in their clinical practice [11]. The inconsistency in these findings concerning the desirability of AI among KSA physicians shows a major gap in the literature. This gap warrants further studies to establish updated statistics on physicians’ attitude toward AI in the KSA and possible factors that influence this attitude. A study that focuses on ethical and practical issues could address this gap by revealing diverse concerns among clinicians.

### Purpose

The purpose of this study was to conduct a cross-sectional survey that would provide updated statistics on physicians’ attitude toward AI with a focus on ethical and practical perspectives among physicians licensed in the KSA. This study will add to existing literature by clarifying the current misunderstanding about physicians’ attitude toward AI, revealing possible contributors toward gaps in AI acceptance, and providing evidence that could guide future initiatives to promote the acceptance of AI among physicians. This study will also contribute toward the KSA’s efforts to become a global leader in AI and other advanced technologies as part of its Vision 2030 initiative [2,6].

## Methods

### Design

A cross-sectional survey was conducted to establish physicians’ attitude toward AI and its integration into the KSA’s health care sector. This design is considered suitable for exploratory studies, such as obtaining participants’ insights about a phenomenon, due to its ease of implementation and collection of numerical data from large samples [12]. The survey was conducted online through self-administered, non–AI-generated questionnaires.

### Population and Sample

This study targeted physicians licensed in the KSA. Physicians are the primary users of clinical decision support systems and are directly impacted by the ethical and practical challenges of AI integration in health care [13]. Given their central role in health care delivery and decision-making, selecting physicians as the study population was appropriate as their attitudes are crucial to the successful adoption and ethical implementation of AI technologies in Saudi Arabia’s health sector.

### Ethical Considerations

This study was approved by the King Abdulaziz University Institutional Review Board, NCBE Registration No: (HA-02-J-008), and adhered to the provisions of the Declaration of Helsinki. This study also respected participants’ autonomy and confidentiality by assuring them that participation was voluntary. Before the survey, all participants provided informed consent electronically via a consent form that outlined the study’s purpose, procedures, confidentiality assurances, and participants’ right to withdraw at any time without penalty. This study posed no risk to participants as it was conducted on physicians through an online survey. No identifying information such as names, addresses, or institutional affiliations was collected to protect privacy and confidentiality. All responses were collected anonymously, and the data were stored on a password-protected device that was accessible only to the principal investigator. No compensation was provided to participants.

### Data Collection and Instruments

A survey of 25 questions was selected for use in this study. This tool was considered suitable for this research as it helped generate valid findings (Cronbach α=0.80) through the use of a sufficiently large sample and maintaining the *P* value at less than .05 [14]. Other than their ease of administration, cost-effectiveness, and ability to reach a large population, questionnaires were selected as they could yield results with high validity and reliability as long as response rates reached or exceeded 40% [12]. The researchers started by creating a questionnaire comprising 7 questions to collect demographic data and an additional 18 questions to collect data on physicians’ attitudes toward AI in health care.

The questionnaire was first disseminated to 10 physicians from the KSA to help identify potential issues with the questions and the methodology. Once the questions were refined, a message was disseminated through posters in local hospitals and online through popular social media platforms (Facebook, X [formerly known as Twitter], Instagram, and WhatsApp) inviting physicians from the KSA to participate in the survey. Participants were assured that their participation would be voluntary and anonymous and that the findings would be used purely for research purposes. A link to an online survey hosted on Qualtrics (Qualtrics International Inc) was included in this message. Participants filled out the survey between February 2025 and April 2025, and the data were downloaded and compiled into spreadsheets. Descriptive statistics, pivot tables, and pivot charts were generated to analyze and interpret the data.

This study adhered to the Checklist for Reporting Results of Internet E-Surveys (CHERRIES), which provides standardized guidance for reporting the design, conduct, and analysis of web-based surveys. Following the CHERRIES framework ensured transparency in describing the survey development process, recruitment procedures, data protection measures, and handling of responses. The completed CHERRIES checklist is provided as a supplementary file (Checklist 1).

The dataset was then exported from Qualtrics into Microsoft Excel and analyzed using descriptive statistics. Inferential analyses were not conducted given the exploratory nature of this study and its primary aim to generate updated descriptive insights into physician attitudes rather than test specific hypotheses. This analytical approach provided a clear overview of prevailing trends and concerns among physicians in the KSA.

## Results

### Demographics

There were a total of 218 surveys filled out during the data collection period. This study had a completion rate of 92.2% (201/218) after removing surveys with blank answers. Table 1 summarizes the demographics of the sample. Women constituted 63.2% (127/201) of the participants and men constituted the remaining 36.8% (74/201). A total of 9.5% (19/201) of the respondents were aged 18 to 25 years, whereas 24.9% (50/201), 28.9% (58/201), 27.9% (56/201), and 9% (18/201) of the respondents were aged 26 to 35 years, 36 to 45 years, 46 to 59 years, and ≥60 years, respectively. Additionally, 12.4% (25/201) of the participants had a PhD, whereas 20.4% (41/201), 9.5% (19/201), and 32.8% (66/201) had a master’s degree, a bachelor’s degree, and an associate degree, respectively. In total, 14.9% (30/201) of the participants had worked as physicians for 3 to 5 years, whereas 30.3% (61/201), 29.4% (59/201), 14.9% (30/201), and 10% (20/201) had worked as physicians for 6 to 10 years, 11 to 20 years, 21 to 30 years, and over 30 years, respectively.

**Table 1.** Demographic characteristics of the physicians who participated in a cross-sectional online survey conducted in Saudi Arabia (N=201).

Education level and age group (years)		Years of experience					
		3‐5 years	6‐10 years	11‐20 years	21‐30 years	Over 30 years	Total
Board certified (n=33, 16.42%)							
	>60	—^a^	—	—	1 (0.50)	8 (3.98)	9 (4.48)
	26‐35	2 (1.00)	—	—	—	—	2 (1.00)
	36‐45	—	1 (0.50)	4 (1.99)	—	—	5 (2.49)
	46‐59	—	—	5 (2.49)	11 (5.47)	1 (0.50)	17 (8.46)
Diploma (n=19, 9.45%)							
	18‐25	15 (7.46)	—	—	—	—	15 (7.46)
	26‐35	3 (1.49)	1 (0.50)	—	—	—	4 (1.99)
Fellow (n=15, 7.46%)							
	>60	—	—	—	—	6 (2.99)	6 (2.99)
	26‐35	1 (0.50)	3 (1.49)	—	—	—	4 (1.99)
	36‐45	—	1 (0.50)	1 (0.50)	—	—	2 (1.00)
	46‐59	—	—	2 (1.00)	—	1 (0.50)	3 (1.49)
Master (n=66, 32.84%)							
	18‐25	1 (0.50)	—	—	—	—	1 (0.50)
	26‐35	—	18 (8.96)	—	—	—	18 (8.96)
	36‐45	—	17 (8.46)	18 (8.96)	1 (0.50)	—	36 (17.91)
	46‐59	—	1 (0.50)	6 (2.99)	4 (1.99)	—	11 (5.47)
MBBS^b^ (n=27, 13.43%)							
	18‐25	3 (1.49)	—	—	—	—	3 (1.49)
	26‐35	5 (2.49)	15 (7.46)	2 (1.00)	—	—	22 (10.95)
	36‐45	—	1 (0.50)	—	—	—	1 (0.50)
	46‐59	—	1 (0.50)	—	—	—	1 (0.50)
PhD (n=41, 20.40%)							
	>60	—	—	—	1 (0.50)	2 (1.00)	3 (1.49)
	36‐45	—	2 (1.00)	9 (4.48)	3 (1.49)		14 (6.97)
	46‐59	—	—	13 (6.47)	9 (4.48)	2 (1.00)	24 (11.94)

a The dashes represent cells with a value of zero.

b MBBS: bachelor of medicine, bachelor of surgery. Now

### Physicians’ Trust in AI Recommendations for Clinical Decision-Making

The findings further revealed that 82.1% (165/201) of the physicians trusted AI-based recommendations in clinical decision-making. This number included 49.3% (99/201) who strongly trusted these systems and 32.8% (66/201) who somewhat partially trusted AI in clinical decision-making. However, 63.7% (128/201) of the physicians believed that AI will always require human oversight in clinical decision-making.

### Physicians’ Perception of AI’s Impact on Clinical Outcomes

Some participants had mixed perceptions of the impact of AI on clinical outcomes. For instance, 56.7% (114/201) of the physicians believed that AI will improve patient outcomes in the long run in the KSA’s health care sector. However, only 5.5% (11/201) of the physicians strongly agreed that AI will improve patient outcomes in the long run, and 52.2% (105/201) of physicians believed that AI could negatively impact physician-patient relationships during the delivery of care.

### Physicians’ Concerns About Ethical Issues Surrounding AI

Most physicians expressed concern about ethical issues surrounding the use of AI in the health care sector. For instance, 54.7% (110/201) were concerned that AI could introduce bias in the diagnosis and treatment of patients. However, 38.8% (78/201) believed that physicians should be held accountable for errors that occur due to the use of AI. Furthermore, 52.2% (105/201) of clinicians expressed concerns about AI systems’ ability to protect patient privacy and data security. As a result, 85.1% (171/201) of the physicians agreed that patients must always be informed when AI systems are involved in diagnosis and treatment. Moreover, 33.8% (68/201) of the respondents cited bias as a key ethical concern, whereas 25.4% (51/201), 20.9% (42/201), and 19.9% (40/201) cited privacy, patient autonomy, and liability, respectively, as some of the ethical concerns that should be addressed to enhance the adoption of AI.

Informed consent was the most dominant concern among respondents. For instance, 70.1% (141/201) of the participants reported facing barriers to obtaining patient consent before the use of AI. On the same note, 89.6% (180/201) agreed that patients’ informed consent was important during AI-based clinical decision-making, whereas 82.1% (165/201) suggested that the informed consent form should mention any clinical recommendations that were made using AI. Furthermore, only 62.2% (125/201) acknowledged that their patients fully understood the consent forms that they signed during treatment. Consequently, 91.5% (184/201) of the physicians always discussed alternatives to AI with their patients before obtaining informed consent to use AI. Many physicians mentioned barriers that hindered the adoption of AI in the KSA’s health care sector. Table 2 illustrates the combinations of barriers that physicians mentioned during the survey.

Participants were presented with a multiple-response (“select all that apply”) question listing common barriers to AI adoption, such as cost, data privacy, lack of training, and resistance to change. Respondents could choose more than one option, and the combinations shown in Table 2 reflect the various sets of barriers selected by each physician.

**Table 2.** Reported barriers to artificial intelligence adoption among physicians participating in a cross-sectional online survey in Saudi Arabia (N=201).

Barriers	Participants, n (%)
Cost	25 (12.4)
Cost and data privacy	6 (3.0)
Cost and resistance to change	2 (1.0)
Data privacy	39 (19.4)
Data privacy and resistance to change	3 (1.5)
Lack of training	46 (22.9)
Lack of training and cost	7 (3.5)
Lack of training, cost, and data privacy	2 (1.0)
Lack of training, cost, data privacy, and resistance to change	5 (2.5)
Lack of training and data privacy	5 (2.5)
Lack of training, data privacy, and resistance to change	5 (2.5)
Lack of training and resistance to change	16 (8.0)
Resistance to change	40 (19.9)

### Physicians’ Perception of AI’s Impact on Efficiency

A total of 76.6% (154/201) of the physicians believed that AI will make health care more efficient. In contrast, 14.4% (29/201) of the respondents believed that AI will complicate health care delivery, whereas 9% (18/201) believed that it will have both positive and negative implications on efficiency. However, all physicians agreed that AI could have widespread applications in the KSA’s health care sector over the next decade. Some of the applications listed by physicians included assisting physicians, automation, and replacement of certain tasks.

### Physicians’ Recommendations on Training Needs for AI

Clinicians offered several recommendations about the training needed to successfully integrate AI into the KSA’s health care sector. A quarter of the respondents (50/201, 25%) mentioned workshops, followed by online courses (47/201, 23.4%), hands-on experience (44/201, 21.9%), and a combination of online courses and hands-on experience (17/201, 8.5%).

## Discussion

### Principal Findings

The data generated from the surveys present an insight into KSA physicians’ perception of the adoption of AI in health care, as well as ethical concerns that these professionals have regarding the application of this technology. Physicians’ recognition of the wide applications of AI is consistent with evidence from the literature. For instance, studies show that AI has vast applications in critical areas such as education, manufacturing, finance, and health care, among others, with the latter being cited as among the latest adopters of this technology [15]. Similar to what KSA physicians reported, AI has been widely applied in the analysis of laboratory tests and diagnostic imaging results, genetic testing diagnosis of diseases, discovery of new treatments, selection of appropriate treatments, robotic surgery, patient monitoring, and various areas of health care that require advanced decision-making [1,15-17]. Patterns in the responses further reflected the ongoing trends in the adoption of AI, which has been considerably higher in high-income countries such as the KSA. Scholars agree that the positive perception may be due to higher access to knowledge, technical expertise, and the infrastructure required to implement the technology [1,18]. The positive perception of AI among KSA physicians may also be explained by the popularization, increased support, and promotion of this technology by the government [2,6]. As such, the adoption of this technology is expected to rise in the coming years.

The hesitancy among KSA physicians to support AI is not surprising considering that this technology is still in its early stages of development. As per the above findings, more physicians from the KSA support the use of AI compared to physicians in specific localities in the country such as Jeddah, the wider Asian region, and parts of the West such as the United States [7,8,10,11]. However, ethical concerns such as patient privacy, data security, liability, patient autonomy, bias in recommendations, and job loss are consistent with concerns documented in previous studies focusing on the KSA, as well as those that investigated the adoption of AI in other parts of the world [7-9,11]. However, the technological revolution being witnessed in the KSA could necessitate the development of AI systems personalized to the needs of the local market to help address these concerns. Nevertheless, addressing the barriers that KSA physicians mentioned, such as training needs, financial constraints, and resistance to change, could ensure that AI gains 100% support among clinicians and optimize the technology’s benefits to patients.

### Study Implications

This study contributes to the KSA’s health care sector as it presents updated statistics on physicians’ perception of AI. These findings will contribute toward the kingdom’s efforts to become a leader in the adoption of advanced technologies such as AI as per its Vision 2030 initiative [2,6]. At the same time, the findings could help other countries striving to adopt AI by highlighting the barriers that they could encounter during the transition and some solutions that they could leverage to achieve success from raising awareness of the benefits of AI and training staff members. Nevertheless, this study underscores the need for comprehensive policy frameworks that balance innovation with ethical responsibility by identifying physicians’ perceived ethical barriers and readiness gaps.

### Study Limitations

This study has some limitations that could affect its application. First, the study’s small sample could affect the validity and generalizability of its findings. Second, the sample was conveniently selected from local hospitals, which could create a risk of selection bias and limit the generalizability of the findings to the entire KSA health care system. Third, the selection of participants from local health care facilities and the use of self-reported surveys raise the risk of self-selection and social desirability bias, which could impact the reliability of the findings. As such, future studies should apply more robust sample selection methods and data collection tools. Fourth, this study suffers from methodological limitations as it was a correlational study that did not establish the actual causes of physicians’ attitude toward AI. Fifth, the application of this study’s findings may be limited, particularly in low- and middle-income nations that experience different challenges from those reported in the KSA. As such, larger studies need to be performed to provide more insights about physicians’ attitude toward AI adoption in health care, factors behind this attitude, and ways to address current concerns. Finally, inferential analyses were not conducted given the exploratory nature of this study and its primary aim to generate updated descriptive insights into physician attitudes rather than test specific hypotheses. As such, future research should apply inferential and multivariate analyses such as logistic regression or structural equation modeling to explore associations among demographic variables, attitudes, and ethical perceptions to facilitate more robust generalizations [12]. Despite these limitations, this study provides a foundational overview that future researchers can build upon while applying more sophisticated analytical models and larger, stratified samples.

### Conclusions

This study explored current attitudes toward AI with a focus on ethical and practical perspectives among physicians licensed in the KSA using a cross-sectional survey design. The findings showed that 82.1% (165/201) of KSA physicians trusted AI-based clinical decision-making and 76.6% (154/201) believed that the technology will improve efficiency in health care delivery. Unfortunately, most physicians (195/201, 97%) reported encountering barriers such as lack of training; high implementation costs; resistance to change; and ethical issues such as privacy, data security, bias in AI-based recommendations, patient autonomy, and liability. Consequently, only 25.9% (52/201) of the physicians reported using AI in the previous year. However, these physicians offered solutions such as informed consent and staff training to address the current barriers to AI adoption in health care.

## Supplementary material

10.2196/79730 Checklist 1 CHERRIES checklist.

## Abbreviations

AI: artificial intelligence
CHERRIES: Checklist for Reporting Results of Internet E-Surveys
KSA: Kingdom of Saudi Arabia
