# Supplementary material for: Understanding Physician Attitudes Toward AI in Clinical Decision-Making: Cross-Sectional Study
Source: JMIR Form Res. 2025 Dec 8;9:e79730. doi: 10.2196/79730 (PMC12685282; doi:10.2196/79730)
Supplement: Checklist 1 [file formative-v9-e79730-s001.docx]

**CHERRIES Checklist: Understanding Physician Attitudes Toward AI in Clinical Decision-Making**

Checklist for Reporting Results of Internet E-Surveys (CHERRIES)

(Adapted from Eysenbach, 2004)

| **Section** | **Item** | **Details from the Study** |
| --- | --- | --- |
| Design | Survey design | A cross-sectional, web-based survey targeting physicians licensed in Saudi Arabia. The survey was self-administered via an online platform (Qualtrics). |
| IRB and Ethics | Ethics approval | The study received approval from King AbdulAziz University Institutional Review Board (IRB) and adhered to the Declaration of Helsinki. |
| IRB and Ethics | Informed consent | Informed consent was obtained electronically. Participants were clearly informed about the purpose of the study, data use, anonymity, and voluntary participation. |
| Development and Pretesting | Development process | The questionnaire was developed by the author, based on literature and previous instruments measuring healthcare providers’ attitudes toward AI. |
| Development and Pretesting | Pre-testing | The survey was pilot-tested with a small group of 5 physicians for clarity, usability, and timing. Minor adjustments were made based on feedback. |
| Recruitment | Recruitment method | Recruitment was conducted through targeted social media posts and physical posters displayed at hospitals across major cities in Saudi Arabia. |
| Recruitment | Inclusion criteria | Physicians must be licensed in Saudi Arabia, currently practicing, and able to read English. |
| Recruitment | Participation | Participants were informed that the survey was open only to physicians and that responses would remain confidential. |
| Survey Administration | Mode and platform | The survey was hosted and conducted on Qualtrics, a secure online survey platform. |
| Survey Administration | Time frame | Data was collected between February and April 2025. |
| Survey Administration | Voluntariness | Participation was entirely voluntary with the right to withdraw at any time. No personally identifiable information was collected. |
| Survey Administration | Incentives | No financial or material incentives were offered. |
| Survey Content and Delivery | Number of items | The survey consisted of 25 questions: 7 demographic and 18 attitude/perception items. |
| Survey Content and Delivery | Adaptive questioning | No adaptive or branching logic was used. All participants answered the same set of questions. |
| Survey Content and Delivery | Mandatory responses | Most questions were required, except for a few optional comment fields. |
| Survey Content and Delivery | Randomization | No randomization of question order was implemented. |
| Survey Content and Delivery | Progress indicator | A progress bar was enabled in the Qualtrics interface. |
| Data Protection and Quality | Anonymity | Responses were completely anonymous. No IP addresses or personal identifiers were collected. |
| Data Protection and Quality | Data storage | Data were securely stored in password-protected files with access restricted to the research team. |
| Data Protection and Quality | Preventing multiple entries | Multiple entries were prevented using Qualtrics settings: a combination of cookies and IP address blocking. |
| Response Rates and Completion | Invitations sent | Approximately 300 physicians were potentially reached via hospital rosters and social media. |
| Response Rates and Completion | Started survey | 218 participants began the survey. |
| Response Rates and Completion | Completed survey | 201 surveys were fully completed and included in the final analysis. |
| Response Rates and Completion | Completion rate | Completion rate = 201 / 218 = 92.2%. |
| Response Rates and Completion | Dropout analysis | 17 incomplete responses were excluded. No significant dropout pattern observed. |
| Analysis | Statistical methods | Data were analyzed using descriptive statistics, pivot tables, and pie charts. |
| Analysis | Handling of incomplete questionnaires | Only fully completed surveys were analyzed to ensure data consistency. |
| Access to Materials | Survey instrument availability | The full survey instrument is available upon request. |
| Access to Materials | Data availability | De-identified data can be made available upon request to qualified researchers. |
